# Low hemoglobin levels and an increased risk of psoriasis in patients with chronic kidney disease

**DOI:** 10.1038/s41598-021-94165-w

**Published:** 2021-07-20

**Authors:** Si-Hyung Lee, Miri Kim, Kyung-Do Han, Ji Hyun Lee

**Affiliations:** 1 Department of Dermatology, Seoul National University College of Medicine, Seoul, Korea; 2 Department of Dermatology, College of Medicine, Yeouido St. Mary’s Hospital, The Catholic University of Korea, Seoul, Korea; 3 Department of Medical Statistics, College of Medicine, The Catholic University of Korea, Seoul, Korea; 4 Department of Dermatology, Seoul St. Mary’s Hospital, College of Medicine, The Catholic University of Korea, 222 Banpo-daero, Seocho-gu, Seoul, 06591 Korea

**Keywords:** Kidney diseases, Skin diseases, Risk factors, Kidney diseases

## Abstract

Chronic diseases, such as chronic kidney disease (CKD), are frequently accompanied by various comorbidities, including anemia, which is considered a surrogate marker of systemic inflammation. Psoriasis is a chronic inflammatory skin disease prevalent in patients with chronic disease. Psoriasis risk in patients with CKD, however, especially in patients with low hemoglobin levels, has never been investigated. In this study, we investigated associations between low hemoglobin levels and psoriasis in patients with CKD using data from the National Health Insurance Service of Korea. During a mean follow-up period of 6.16 ± 1.02 years, psoriasis was recorded in 13,803 patients with CKD (2.39% of CKD patients). The cumulative incidence of psoriasis was significantly higher in CKD patients with anemia (hemoglobin levels < 13 g/dL in men and < 12 g/dL in women) than those without. In multivariate-adjusted Cox proportional hazards regression models, the risk of psoriasis was significantly higher in anemic CKD patients than nonanemic CKD patients (hazard ratio [HR] 1.136, 95% CI 1.089–1.185, p < 0.001). Additionally, we noted that the incidence of psoriasis decreased with increasing hemoglobin levels in CKD patients (HR 0.953, 95% CI 0.942–0.965, p < 0.001). Altogether, our findings indicate that low hemoglobin levels are significantly related to psoriasis risk in patients with CKD. Further study is required to elucidate whether low hemoglobin levels have an impact on the development of psoriasis or are merely a surrogate marker of psoriasis risk in patients with CKD.

## Introduction

Anemia is a common occurrence in chronic diseases, such as chronic kidney disease (CKD), chronic obstructive pulmonary disease, heart disease, etc., and in healthy older adults^1–4^. In chronic diseases, anemia has been shown to be closely associated with poor quality of life, higher mortality, and various comorbidities, including cardiovascular disease^3,5–8^. In patients with CKD in particular, anemia has been found to be associated with symptoms of fatigue, dyspnea, and exercise intolerance, as well as with higher rates of hospitalization and mortality, and treatments for anemia appear to have beneficial effects on cardiovascular comorbidity and hospitalization and mortality rates^6,7,9,10^.

Psoriasis is chronic inflammatory skin disease in which IL23 and T helper 17 (Th17) cells play a central role^11^. Cumulative evidence suggests that psoriasis is not merely a cutaneous disease, but a systemic inflammatory disease accompanied by various comorbidities, including psoriatic arthritis, inflammatory bowel disease, and cardiovascular disease^12^. Recently, population-based cohort studies have indicated that psoriasis is an independent risk factor of CKD^13–15^. However, to the best of our knowledge, studies have yet to evaluate psoriasis risk in patients with CKD. Since anemia is common in chronic diseases, such as CKD, in this study, we sought to evaluate potential relationships between anemia and psoriasis risk in CKD patients though a population-based cohort study.

## Results

### Clinical characteristics of the study population

Clinical characteristics of CKD patients with or without anemia are summarized in Table 1. Of the 576,461 CKD patients, anemia was present in 108,304 (18.8%). Mean age (63.37 ± 14.39 vs. 56.13 ± 15.08 years) and the percentages of male patients (28.95 vs. 43.96%), urban residents (47.28 vs. 49.37%), patients with low income (23.1 vs. 19.37%), current smokers (8.21 vs. 17.03%), heavy drinkers (1.67 vs. 4.29%), and patients with regular physical activity (38.52 vs. 49.18%) were significantly different between the CKD patients with and without anemia. Interestingly, the prevalences of hypertension (59.32 vs. 43.3%), dyslipidemia (33.6 vs. 29.52%), stroke (4.27 vs. 2.53%), and heart disease (11 vs. 6.06%) were significantly higher in CKD patients with anemia than those without anemia. In contrast, the prevalence of diabetes (84.7 vs. 73.45%) was significantly higher in CKD patients without anemia. CKD patients with anemia exhibited significantly lower height (156.89 ± 8.55 vs. 160.86 ± 9.29 cm), weight (57.92 ± 9.91 vs. 62.8 ± 10.93 kg), waist circumference (80.61 ± 9.2 vs. 81.93 ± 9.1 cm), body mass index (BMI, 23.48 ± 3.26 vs. 24.2 ± 3.22 kg/m^2^), diastolic blood pressure (75.95 ± 10.53 vs. 77.37 ± 10.12 mmHg), and total cholesterol (191.38 ± 40.89 vs. 201.05 ± 39.05 mg/dL). Also, systolic blood pressure (126.32 ± 17.33 vs. 125.64 ± 15.92 mmHg), fasting glucose (104.82 ± 32.41 vs. 101.78 ± 27.15 mg/dL), and glomerular filtration rate (45.5 ± 16.11 vs. 42.08 ± 21.21 ml/min/1.73m^2^) were significantly higher in CKD patients with anemia than in their non-anemic counterparts.

**Table 1:** Baseline characteristics of chronic kidney disease patients according to the presence of anemia.

	Anemia*		*P* value
	Absent	Present	
	n = 468,157	n = 108,304	
Age, years	56.13 ± 15.08	63.37 ± 14.39	< 0.0001
Men, n (%)	205,798 (43.96)	31,352 (28.95)	< 0.0001
Urban resident, n (%)	231,148 (49.37)	51,201 (47.28)	< 0.0001
Low income, n (%)	90,677 (19.37)	25,013 (23.1)	< 0.0001
Current smoker, n (%)	79,723 (17.03)	8891 (8.21)	< 0.0001
Heavy drinker, n (%)	20,080 (4.29)	1805 (1.67)	< 0.0001
Regular physical activity, n (%)	230,262 (49.18)	41,716 (38.52)	< 0.0001
Diabetes, n (%)	396,514 (84.7)	79,546 (73.45)	< 0.0001
Hypertension, n (%)	202,724 (43.3)	64,244 (59.32)	< 0.0001
Dyslipidemia, n (%)	138,223 (29.52)	36,389 (33.6)	< 0.0001
Stroke, n (%)	8938 (2.53)	3444 (4.27)	< 0.0001
Heart disease, n (%)	21,410 (6.06)	8879 (11)	< 0.0001
Height, cm	160.86 ± 9.29	156.89 ± 8.55	< 0.0001
Weight, kg	62.8 ± 10.93	57.92 ± 9.91	< 0.0001
Waist circumference, cm	81.93 ± 9.1	80.61 ± 9.2	< 0.0001
BMI, kg/m^2^	24.2 ± 3.22	23.48 ± 3.26	< 0.0001
DBP, mmHg	77.37 ± 10.12	75.95 ± 10.53	< 0.0001
SBP, mmHg	125.64 ± 15.92	126.32 ± 17.33	< 0.0001
Fasting glucose, mg/dL	101.78 ± 27.15	104.82 ± 32.41	< 0.0001
Total cholesterol, mg/dL	201.05 ± 39.05	191.38 ± 40.89	< 0.0001
GFR, ml/min/1.73m^2^	42.08 ± 21.21	45.5 ± 16.11	< 0.0001

Data are presented as means ± standard deviations or numbers and percentages.

*BMI* body mass index; *DBP* diastolic blood pressure; *SBP* systolic blood pressure; *GFR* glomerular filtration rate.

*Anemia was defined as a hemoglobin level of < 13 g/dL in men and < 12 g/dL in women.

### Relationship between hemoglobin levels and psoriasis in CKD patients

**Figure 1:**
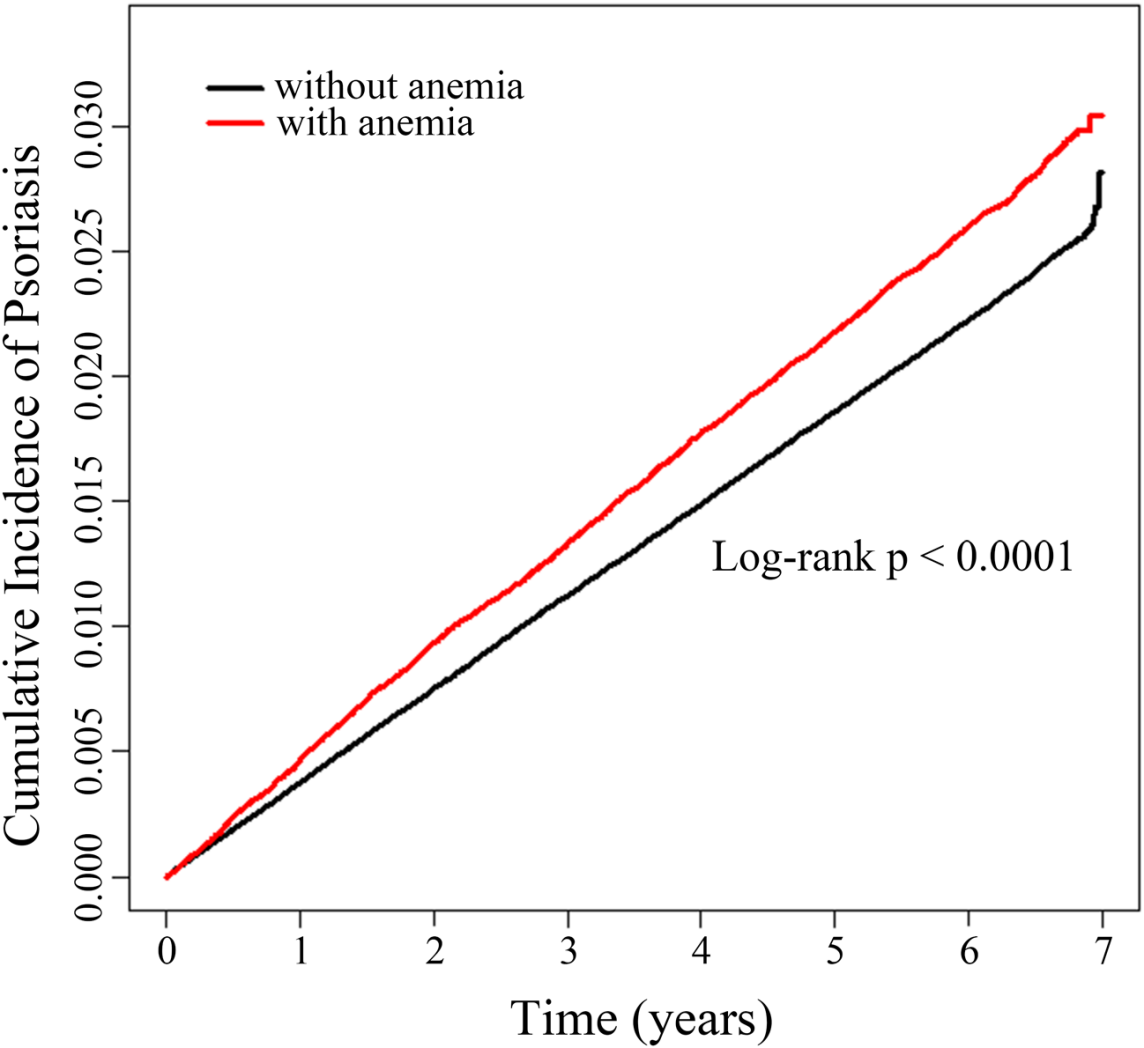
Cumulative incidences of psoriasis according to the presence of anemia in patients with chronic kidney disease.

During a mean follow-up period of 6.16 ± 1.02 years, psoriasis developed in 13,803 of the 576,461 CKD patients (2.39%). Interestingly, the CKD patients with anemia showed a higher cumulative incidence of psoriasis than the CKD patients without anemia (log-rank p < 0.0001, Fig. 1). Next, we evaluated the risk of psoriasis in three models adjusting for different confounding variables (see Materials and Methods). As shown in Table 2, CKD patients with anemia exhibited higher risks of psoriasis in all three models (model 1, hazard ratio [HR] 1.168, 95% CI 1.121–1.218; model 2, HR 1.136, 95% CI 1.089–1.185; model 3, HR 1.109, 95% CI 1.062–1.158).

**Table 2:** Hazard ratios for psoriasis according to the presence and severity of anemia among patients with chronic kidney disease.

	Person years	HR (95% CI)		
		Model 1	Model 2	Model 3
Anemia*				
No (n = 10,978)	2,912,237.2	1 (reference)	1 (reference)	1 (reference)
Yes (n = 2825)	641,507.4	1.168 (1.121–1.218)	1.136 (1.089–1.185)	1.109 (1.062–1.158)
*P* value		< 0.0001	< 0.0001	< 0.0001
**Hemoglobin quintiles**				
Q1 (n = 3172)	690,432.9	1 (reference)	1 (reference)	1 (reference)
Q2 (n = 2657)	676,810.07	0.854 (0.811–0.9)	0.88 (0.836–0.927)	0.903 (0.858–0.952)
Q3 (n = 2786)	754,565.8	0.804 (0.764–0.846)	0.871 (0.827–0.917)	0.891 (0.846–0.938)
Q4 (n = 2504)	687,743.26	0.793 (0.752–0.835)	0.873 (0.827–0.921)	0.889 (0.842–0.939)
Q5 (n = 2684)	744,192.57	0.785 (0.746–0.826)	0.867 (0.823–0.914)	0.877 (0.831–0.926)
*P* for trend		< 0.0001	< 0.0001	< 0.0001
**Hemoglobin of 1 g/dL**	0.995 (0.985–1.006)	0.953 (0.942–0.965)	0.957 (0.946–0.969)	
*P* value	< 0.0001	< 0.0001	< 0.0001	

Model 1 was not adjusted for any variable. Model 2 was adjusted for age and sex. Model 3 was adjusted for age, sex, body mass index, smoking status, alcohol consumption, physical activity, income, glomerular filtration rate, diabetes, hypertension, and dyslipidemia.

*Anemia was defined as a hemoglobin level of < 13 g/dL in men and < 12 g/dL in women.

**Figure 2:**
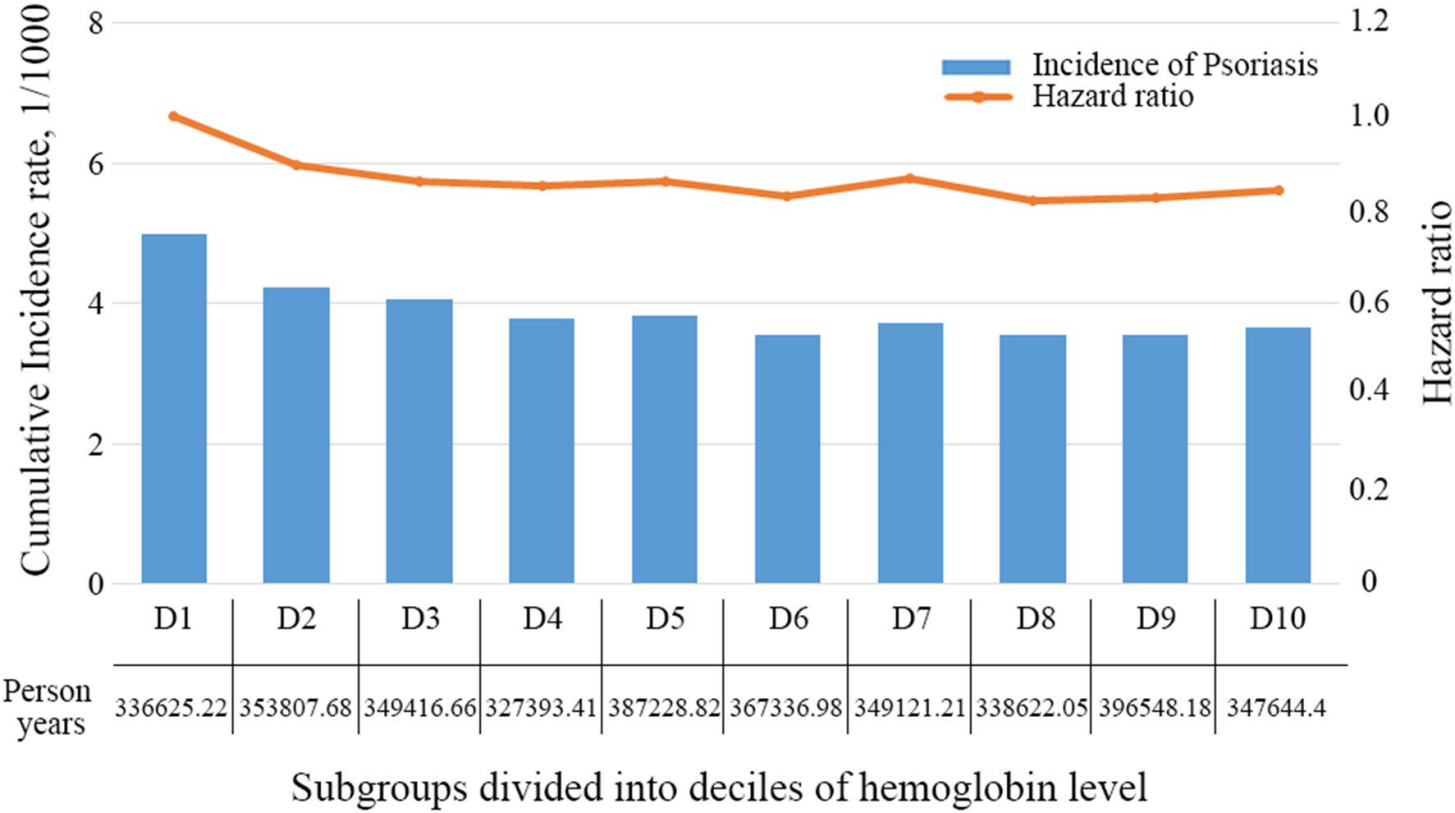
Cumulative incidences and hazard ratios of psoriasis according to deciles of hemoglobin levels in chronic kidney disease patients.

Also, we assessed psoriasis risk according to quintiles of hemoglobin levels (Q1-Q5). The mean hemoglobin levels were 11.4 ± 1.1 g/dL in Q1, 12.9 ± 0.9 g/dL in Q2, 13.5 ± 0.9 g/dL in Q3, 14.2 ± 1.0 g/dL in Q4, and 15.1 ± 1.2 g/dL in Q5. Compared to lowest hemoglobin quintile (Q1), the risk of psoriasis decreased with increases in hemoglobin and followed a linear trend across hemoglobin quintiles. This finding was consistent across models 1, 2, and 3 (Table 2). Moreover, in model 3, the cumulative incidences and HRs of psoriasis showed similar trends across subgroups divided into deciles of hemoglobin levels (Fig. 2). As expected, psoriasis risk decreased significantly with an increase in hemoglobin, and this finding was consistent in models 1, 2, and 3 (Table 2).

## Discussion

Psoriasis is a chronic inflammatory skin disease accompanied by systemic inflammation, therefore, several comorbidities, such as cardiovascular disease, inflammatory bowel disease and psoriatic arthritis, are associated with psoriasis^12^. In addition, there have been several reports regarding the disease with a higher risk of psoriasis. In addition to diseases shared immunopathologic connection with psoriasis, such as Crohn’s disease, several diseases, which are not mainly caused by activation of Th17 cell-mediated pathway, such as rheumatoid arthritis, celiac disease, and chronic periodontitis, also revealed a higher risk of psoriasis^16–19^. This finding might be partially explained by inflammatory microenviroment in these disease might provide favorable conditions for the development of psoriasis.

Chronic diseases, such as CKD, are frequently accompanied by inflammation^20^, and chronic inflammatory status in CKD has been shown to be associated with several underlying factors, increased infection rates, elevated cytokine levels, and other cardiovascular comorbidities^21^. Due to impaired renal function, patients with CKD typically present with increased serum levels of inflammatory molecules, including interleukin-1 (IL-1), IL-6, and C-reactive protein^22,23^. Based on various clinical and experimental findings, researchers have suggested that inflammation in patients with CKD is not only a predisposing factor for cardiovascular comorbidities, but also an aggravating factor for CKD^24,25^.

The clinical significance of chronic inflammation in CKD is further emphasized by its causative effect on anemia. In patients with chronic disease, such as CKD, chronic heart disease, and chronic pulmonary disease, persistent systemic inflammation restricts erythropoiesis and hinders erythrocyte survival through inflammatory cytokines^26,27^. In this study, we found that anemia and hemoglobin levels were closely associated with psoriasis risk in CKD patients. The association between hemoglobin levels and psoriasis was independent of age, sex, BMI, current smoking status, alcohol consumption, physical activity, income level, glomerular filtration rate, diabetes, hypertension, and dyslipidemia. Since the development of anemia and resistance to treatment for anemia are induced by systemic inflammation in CKD, anemia may be a surrogate marker of psoriasis risk. However, further investigation of the effect of anti-inflammation treatment on the development of psoriasis in CKD patients would be required to address this hypothesis.

Hypoxia-inducible factors (HIFs), which are transcription factors required for cellular adaptation of hypoxia, play a crucial role in pathogenesis of CKD, as well as CKD-associated comorbidities, through multiple mechanisms^28^. Interestingly, HIFs have also been found to be upregulated in the lesional skin of psoriasis patients^29^. Meanwhile, several studies have indicated that HIF-1α plays a role in the development of psoriasis: HIF-1α induces the expression of vascular endothelial growth factor (VEGF), which is also upregulated in psoriatic lesions, and the activation of VEGF induces psoriasis like lesions, possibly through induction of angiogenesis^29,30^. In addition, HIF-1α is involved in the differentiation of T helper cell 17 (Th17), a key cellular component in the immunopathogenesis of psoriasis, and in the production of IL-17^31^. Interestingly, research suggests that a Th17 immune response could induce anemia through suppression of the hematopoietic system^32^. Moreover, studies have shown that Th17 cells and IL17 are important in renal injury induced by high salt intake or ischemia/reperfusion injury^33,34^. Accordingly, we suspect that the development of both CKD and anemia could be accompanied by activation of a Th17 immune response associated with psoriasis development, making psoriasis a comorbidity of CKD, especially in anemic individuals.

Psoriasis is well known for its association with poor quality of life^35^. Moreover, in CKD patients with anemia, who likely have severely impaired renal function, systemic treatment of psoriasis, such as that with cyclosporine or methotrexate, is limited. Therefore, in CDK patients with anemia, we propose that controlling the risk of psoriasis by effectively managing anemia and systemic inflammation could help with limiting psoriasis and its adverse effects on quality of life. Since inflammatory cytokines underlie the development of anemia and resistance to erythropoietin treatment, anti-inflammatory treatment could be beneficial in the treatment of anemia and the prevention of psoriasis in CKD patients^36^. Indeed, research has shown that pentoxifylline, which inhibits the production of T-helper cell-derived cytokines, improves anemia in CKD patients^37,38^.

In conclusion, we found that low hemoglobin levels were significantly related to an increased risk of psoriasis in CKD patients. Although it is unclear whether the observed increased risk in psoriasis is directly affected by an anemic condition or is merely a surrogate marker of systemic inflammation underlying the development of psoriasis, proactive treatment for inflammation might play crucial role in managing both anemia and psoriasis in CKD patients.

## Materials and methods

### Data source and study population

The dataset used in this retrospective cohort study was provided by the National Health Insurance Service (NHIS) of Korea and included information on age, sex, socioeconomic variables, disease diagnoses based on the ICD-10 Clinical Modification (ICD-10-CM), medical treatments and procedures, and health examination results recorded in inpatient and outpatient claims for the majority of the South Korean population^39^. The study population was recommended to undergo standardized medical examinations every 2 years by the National Health Insurance Corporation.

**Figure 3:**
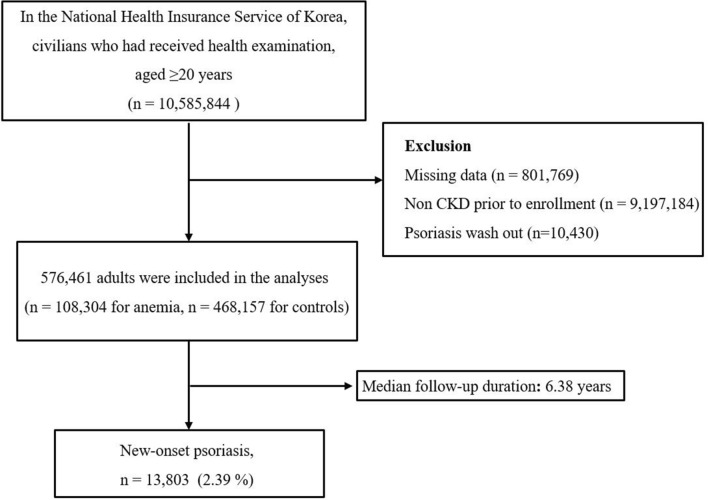
Flow chart of enrolled population.

In the NHIS database, individuals with chronic kidney disease (CKD) were identified using the International Classification of Disease, 10th revision code N18. CKD patients with a confirmed ICD-10-CM diagnostic code given during health screening from January 2009 to December 2009 were enrolled in this study. CKD patients diagnosed with psoriasis before this study period were excluded (Fig. 3). This study was approved by the Institutional Review Board of the Korean National Institute for Bioethics Policy (NHIS-2019–01-076) and the Ethics Committee of Seoul St. Mary’s Hospital, the Catholic University of Korea (KC19ZESI 0236). All data were anonymized and de-identified, and informed consent was waived by the Institutional Review Board of the Korean National Institute for Bioethics Policy. This study was performed in accordance with the principles of the Declaration of Helsinki.

### Measurement and definition of covariates

Medical records included measurements of height, weight, waist circumference, and blood pressure; laboratory test results for fasting glucose, total cholesterol, and glomerular filtration rate; and past medical history. Information on health-related behaviors, such as smoking, drinking, and physical activity, were obtained using a standardized self-reporting questionnaire. Smoking status was categorized as nonsmoker, former smoker, and current smoker. Heavy drinkers were defined as individuals who consumed ≥ 30 g of alcohol per day^40^. Regular physical activity was categorized as strenuous physical activity for ≥ 20 min at least three times per week or moderate physical activity for ≥ 30 min at least five times per week. Household income level in the lower 25% was defined as low income.

### Study design

To examine the relationship between anemia and psoriasis risk in CKD patients, the patients were subdivided into subgroups according to the presence of anemia and the severity thereof. In this study, anemia was defined as a hemoglobin level of < 13 g/dL in men and < 12 g/dL in women. In addition, CKD patients were subdivided into five and 10 groups according to quintiles and deciles of hemoglobin levels, respectively. Among these subgroups, we identified patients newly diagnosed with psoriasis (ICD-10-CM code, L40) over a mean follow-up period of 6.16 years through claims data.

### Statistical analysis

The baseline characteristics of the CKD patients are presented as means with standard deviations or numbers and percentages. The cumulative incidences of psoriasis according to the presence of anemia in CKD patients were depicted using Kaplan–Meier curves, and the log-rank test was used to analyze differences therein between groups. To analyze the presence or severity of anemia in relation to psoriasis risk, multivariate-adjusted Cox proportional hazards regression models were used in three different ways: Model 1 was not adjusted for any variable. Model 2 was performed adjusting for age and sex. BMI, smoking status, alcohol consumption, physical activity, income, glomerular filtration rate, diabetes, hypertension, and dyslipidemia were further adjusted in model 3. Variables included in these models were chosen according to it’s clinical in the development of psoriasis and statistical significance in our study. Compared to controls (CKD patients without anemia and in the lowest hemoglobin quintile), hazard ratios (HRs) and 95% CIs were calculated. Statistical significance was defined as a two-sided *P* value less than 0.05. All statistical analyses were performed using SAS software (ver. 9.4; SAS Institute, Cary, NC, USA) and R programming (version 3.1.0; The R Foundation for Statistical Computing, Vienna, Austria).
